# Gp120 V5 Is Targeted by the First Wave of Sequential Neutralizing Antibodies in SHIV_SF162P3N_-Infected Rhesus Macaques

**DOI:** 10.3390/v10050262

**Published:** 2018-05-16

**Authors:** Manxue Jia, Hong Lu, Xiang-Peng Kong, Cecilia Cheng-Mayer, Xueling Wu

**Affiliations:** 1 Aaron Diamond AIDS Research Center, Affiliate of The Rockefeller University, New York, NY 10016, USA; mjia@adarc.org (M.J.); lhong@adarc.org (H.L.); cmayer@adarc.org (C.C.-M.); 2 School of Medicine, New York University, New York, NY 10016, USA; xiangpeng.kong@med.nyu.edu

**Keywords:** HIV vaccine, SHIV, rhesus macaque, neutralizing antibody

## Abstract

Simian-human immunodeficiency virus (SHIV) infection provides a relevant animal model to study HIV-1 neutralization breadth. With previously identified SHIV_SF162P3N_ infected rhesus macaques that did or did not develop neutralization breadth, we characterized the transmitted/founder viruses and initial autologous/homologous neutralizing antibodies in these animals. The plasma viral load and blood CD4 count did not distinguish macaques with and without breadth, and only one tested homologous envelope clone revealed a trend for macaques with breadth to favor an early homologous response. In two macaques with breadth, GB40 and FF69, infected with uncloned SHIV_SF162P3N_, multiple viral variants were transmitted, and the transmitted variants were not equal in neutralization sensitivity. The targets of initial autologous neutralizing antibodies, arising between 10 and 20 weeks post infection, were mapped to N462 glycan and G460a in gp120 V5 in GB40 and FF69, respectively. Although it is unclear whether these targets are related to later neutralization breadth development, the G460a target but not N462 glycan appeared more common in macaques with breadth than those without. Longitudinal plasmas revealed 2–3 sequential waves of neutralizing antibodies in macaques with breadth, implicating that 3 sequential envelope variants, if not more, may be required for the broadening of HIV-1 neutralizing antibodies.

## 1. Introduction

Elicitation of HIV-1 broadly neutralizing antibody (bnAb) responses is desired for an effective vaccine [1,2,3]; however, current HIV-1 vaccines cannot induce such responses, despite the fact that a fraction of HIV-1 infected individuals develop bnAbs after 2–3 years of infection [4,5,6]. To gain an understanding of bnAb development during the course of infection, a few studies have longitudinally tracked bnAb lineages [7,8,9,10,11] or development [12,13] in HIV-1 infected individuals, showing that the bnAb lineages and their development have always started from early times in infection when autologous but not heterologous neutralizing antibodies are detected. Therefore, there is an interest to characterize the early autologous neutralizing antibody responses to link them to later bnAb development in HIV-1 infection.

Studies of several clade B infected individuals have reported the emergence of autologous neutralizing antibodies between 2 and 6 months post infection (mpi) [14,15]. Similarly, studies of one clade A and several clade C infected individuals also noted the timing of 2–6 mpi for autologous neutralizing antibodies, and that these antibodies target the regions of gp120 V1V2, α2 helix of C3, or C3, and other undefined regions in V3–V5 [16,17,18]. Monoclonal antibodies directed to V1V2 and α2 of C3 have been isolated from three (one clade A and two clade C) infected individuals and shown autologous strain-specific neutralizing activity [18,19,20]. Hence, these studies have provided a basic understanding of the dynamics and targets of the initial autologous neutralizing antibodies in HIV-1 clade A, B and C infected individuals.

As HIV-1 vaccine candidates rely on nonhuman primates (NHP) for preclinical tests [21], it is also important to characterize NHP antibody responses to the HIV-1 envelope (Env), and the most relevant model has been the simian-human immunodeficiency virus (SHIV) infected rhesus macaques, especially with SHIVs bearing the Env sequences that elicited bnAbs in humans [22]. To date, bnAbs against HIV-1 tier 2 isolates have rarely been detected in SHIV-infected macaques [6,23,24,25], and the neutralizing antibody responses in these macaques have not been longitudinally characterized. We have previously screened 13 viremic macaques infected intrarectally with uncloned SHIV_SF162P3N_ [26] or its derivative molecular clones [27] and determined that 5 macaques developed HIV-1 neutralization breadth [6]. Taking the advantage of known infection time, route and virus, as well as frequently sampled time points, we are interested in understanding the bnAb development in these animals. To assess potential factors that distinguish these macaques from those that did not develop neutralization breadth, we also included 6 SHIV_SF162P3N_ infected but “no breadth” macaques [6] that mounted autologous or homologous neutralizing antibody responses for comparison. Here we focused on the characterization of the dynamics and targets of the initial autologous neutralizing antibody responses in these animals and then compared them to those reported in HIV-1 infected humans.

## 2. Materials and Methods

### 2.1. Ethics Statement

The rhesus plasma samples analyzed in this study were from macaques described in previous studies [27,28,29,30]. All of the Chinese and Indian rhesus macaques received a single dose intrarectal challenge of uncloned SHIV_SF162P3N_ [26] or its derivative molecular clones [27]. The clade B R5 pathogenic SHIV_SF162P3N_ was recovered from a SHIV_SF162_ passage 3 macaque T353 at the time of euthanasia after 66 weeks of infection [26]. All of the macaque specimens were collected at the Tulane National Primate Research Center in compliance with its Guide for the Care and Use of Laboratory Animals and under protocols approved by the Institutional Animal Care and Use Committee (IACUC) (animal welfare assurance number A3081-01, approval date 8 October 2012).

### 2.2. Cells, Plasmids and Viruses

The TZM-bl cells [31] and HIV-1 SG3Δenv backbone [14,31] were obtained from the NIH AIDS Reagent Program, as contributed by John Kappes and Xiaoyun Wu. The SHIV Env-pseudoviruses were prepared by co-transfecting 293T cells (ATCC, Manassas, VA, USA) with the SHIV *rev/env* plasmids and the HIV-1 SG3Δenv backbone.

### 2.3. SHIV env Single Genome Amplification and Cloning

The SHIV *env* gene of the infected macaques GB40 and FF69 were amplified and cloned using the single genome amplification (SGA) method described previously [32,33,34]. Briefly, 140 μL plasma from selected time points was used to extract viral RNA using the QIAamp viral RNA mini kit (Qiagen, Valencia, CA, USA). Reverse transcription (RT) was carried out in a total volume of 100 μL, including 60 μL viral RNA, 1.25 μL antisense primer SH51 [35] at 20 μM, 5 μL dNTP (each at 10 mM), 20 μL 5× first-strand buffer, 5 μL dithiothreitol (DTT) at 100 mM, 5 μL RNaseOUT, and 5 μL SuperScript III (Invitrogen, Carlsbad, CA, USA). The reaction was incubated at 50 °C for 60 min, followed by 55 °C for an additional 60 min and 70 °C for 15 min. The cDNA was titrated to a single copy where PCR-positive wells constitute about 30% of total reactions. Nested PCRs were carried out in 20 μL consisting of 2 μL 10× buffer, 0.8 μL MgSO_4_, 0.4 μL dNTP (each at 10 mM), 0.2 μL of each primer at 20 μM, 0.1 μL Platinum Taq High Fidelity polymerase (Invitrogen), and 1 μL template DNA. The 1st-round PCR primers were envB5out [34] and SH51 [35]; the 2nd-round PCR primers were envB5in [34] and SH44 [35]. The cycler parameters were 94 °C for 2 min, followed by 35 cycles (45 cycles for 2nd-round) of 94 °C for 15 s, 55 °C for 30 s, and 68 °C for 4 min, and then a final extension of 68 °C for 10 min. The PCR amplicons were subjected to direct Sanger sequencing; all sequencing chromatograms were inspected in Sequencher 5.4 (Gene Codes, Ann Arbor, MI, USA) for mixed bases (double peaks), which would be evidence of priming from more than one template or PCR errors; any sequence with evidence of double peaks was excluded. The sequences of interest were codon-aligned with ClustalW built in BioEdit (http://www.mbio.ncsu.edu/bioedit/bioedit.html) and then manually checked. The nucleotide distance matrix was calculated by DNAdist in BioEdit. The neighbor-joining tree was constructed using MEGA6 [36] (http://www.megasoftware.net) with 1000 bootstraps and then displayed with Dendroscope version 3.5.9 (http://ab.inf.uni-tuebingen.de/data/software/dendroscope/download/welcome.html). Representative *env* sequences were reamplified from the 1st-round PCR containing the full-length *rev/env* genes and cloned into pcDNA3.1D TOPO (Invitrogen) for expression.

### 2.4. Viral Neutralization Assay

Viral neutralization was measured using single round infection of TZM-bl cells with Env-pseudoviruses as described [37,38]. Briefly, 50 μL of antibody-virus mixture was incubated at 37 °C for 30 min in duplicate wells before the addition of TZM-bl cells. To keep assay conditions constant, sham medium was used in place of antibody in control wells. Infection levels were determined after two days with Bright-Glo luciferase assay system (Promega, Madison, WI, USA). Neutralization curves were fit by nonlinear regression using a 5-parameter hill slope equation built in Prism 6.0 (GraphPad Software, La Jolla, CA, USA). The plasma reciprocal dilutions required to inhibit infection by 50% was reported as ID_50_ titers.

### 2.5. Site-Directed Mutagenesis

The mutations of interest were introduced with the TagMaster site-directed mutagenesis kit (GM Biosciences, Frederick, MD, USA). The template plasmid was amplified with each forward and reverse primer containing the mutation of interest, and then the Top10 chemical-competent cells (Invitrogen) were transformed with the PCR product. Colonies were screened for the presence of the desired mutation by DNA sequencing, followed by sequencing of the entire *rev/env* region for the final plasmid prep.

### 2.6. Statistical Analysis

Unpaired Mann-Whitney test and contingency table with Fisher’s exact test implemented in Prism 6.0 was used to compare the plasma viral load, CD4 count and dynamics of antibody response between macaques with and without neutralization breadth. *p* values of 0.05–0.1 were considered trends, and *p* values of <0.05 were considered statistically significant.

### 2.7. Nucleotide Sequence Accession Numbers

The full-length SHIV *env* nucleotide sequences reported in this study have been deposited to GenBank under accession numbers MH256668–MH256800.

## 3. Results

### 3.1. Comparisons between SHIV-Infected Macaques with and without HIV-1 Neutralization Breadth

**Figure 1:**
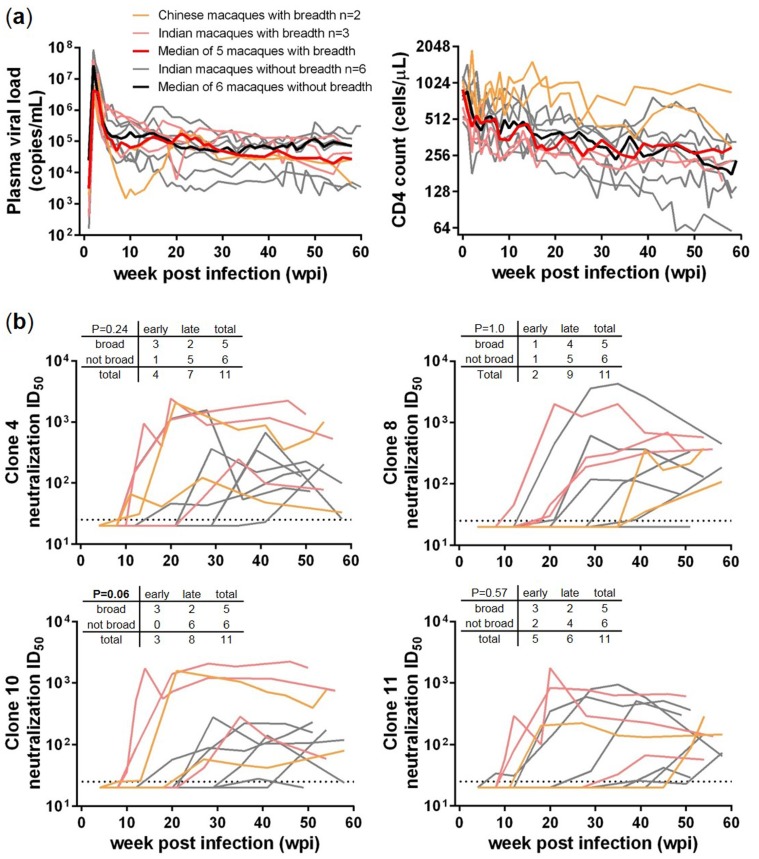
Comparisons between SHIV_SF162P3N_ infected rhesus macaques with (*n* = 5, light orange/red) and without (*n* = 6, grey) HIV-1 neutralization breadth. (**a**) Longitudinal plasma viral load and CD4 count, with bold red and black lines indicating the median values of the two macaque groups; (**b**) Longitudinal homologous neutralization ID_50_ titers against 4 SHIV_SF162P3N_ inoculum-derived Env-pseudoviruses, clones 4, 8, 10 and 11. The early (before 20 wpi) and late (after 20 wpi) neutralization responses were compared between the two macaque groups in a contingency table included in each neutralization plot, with Fisher’s exact test *p* values indicated.

Previously, using the criteria of neutralizing 5 or more out of 10 tested HIV-1 Env isolates, with at least one ID_50_ > 100, 5 SHIV_SF162P3N_ infected rhesus macaques were determined to have bnAbs against HIV-1, including 2 Chinese rhesus macaques GB40 and GL26, and 3 Indian rhesus macaques FF69, FD83 and DD80 [6]. To understand the potential factors that distinguish them from those that did not develop neutralization breadth, we included 6 “no breadth” Indian rhesus macaques that mounted autologous or homologous responses—namely EN13, EP30, DP85, DP87, FF59 and FF94 [6], for comparison (Figure 1). Comparing the plasma viral load and blood CD4 count for up to 60 weeks post infection (wpi), the two group median values largely overlapped (Figure 1a), and no significant difference was detected between the two groups using unpaired Mann-Whitney test. Therefore, plasma viral load and blood CD4 count did not seem to distinguish the two groups.

We next compared the dynamics and plasma neutralization ID_50_ titers against 4 Env clones derived from the SHIV_SF162P3N_ inoculum, clones 4, 8, 10 and 11 (Figure 1b). In general the dynamics of plasma neutralization against these homologous clones segregated into an early (before 20 wpi) and a late (after 20 wpi) stage, and the plasma neutralizing ID_50_ titers segregated from high (>1000) to low or moderate (~100). We used contingency tables with Fisher’s exact test to compare the dynamics of neutralization response between the two groups. No significant difference was detected for clones 4, 8 and 11, but the response to clone 10 revealed a trend (*p* = 0.06) for macaques with breadth to favor an early response. Therefore, though early development of a strong homologous neutralization response might favor the development of neutralization breadth later on, the association was not always there and thus lacked predictive power.

### 3.2. Transmitted/Founder env Sequences in SHIV-Infected Macaques

To characterize autologous neutralizing antibody responses in macaques infected with uncloned SHIV_SF162P3N_ [26], we selected animals GB40 and FF69 that later developed HIV-1 neutralization breadth and examined by SGA the *env* sequences of their transmitted/founder (T/F) viruses. From the week 2 (w2) plasmas with peak viremia, we obtained a total of 22 and 57 *env* sequences from GB40 and FF69, respectively (Table 1). The maximum distance among GB40_w2 *env* sequences was 2.12%, with a mean distance of 0.74%. Likewise, the maximum distance among FF69_w2 *env* sequences was 2.17%, with a mean distance of 1.01%. These data indicated that the T/F viruses in these animals were not homogeneous. We also obtained 54 *env* sequences from the FF69_w8 plasma, which had a maximum distance of 2.33% and a mean distance of 1.15%, both slightly increased from the FF69_w2 *env* sequences (Table 1).

**Figure 2:**
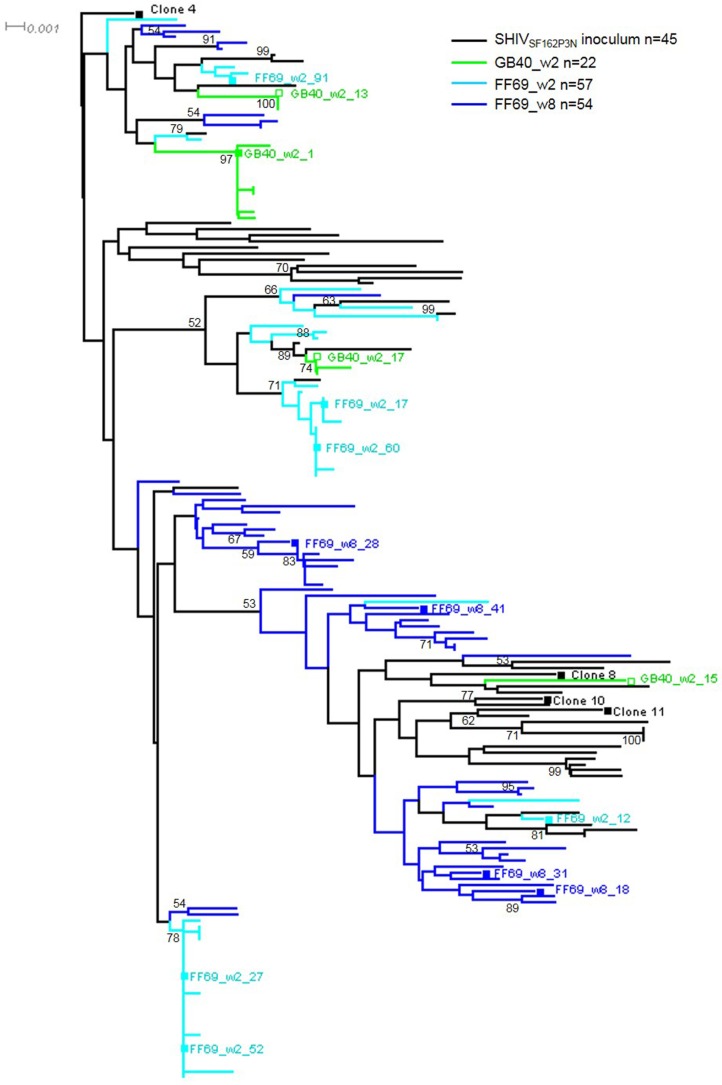
Transmitted/founder (T/F) viruses in SHIV_SF162P3N_ infected macaques GB40 and FF69. The color-coded neighbor-joining tree shows the gp120 V1-V5 nucleotide sequences from the SHIV_SF162P3N_ inoculum, GB40_w2 plasma, FF69_w2 and FF69_w8 plasmas. The tree is rooted at the SHIV_SF162P3N_ inoculum-derived clone 4, with the scale bar indicated and bootstrap values >50 shown at the corresponding branch nodes. The cloned full-length *env* sequences are indicated with a square symbol and labeled with the clone number.

With a total of 45 published V1–V5 sequences from the SHIV_SF162P3N_ inoculum [26], we assessed the GB40_w2, FF69_w2 and FF69_w8 V1–V5 sequences in a neighbor-joining tree, rooted at clone 4 (Figure 2). The 22 GB40_w2 sequences revealed three clusters, with a dominant cluster of 13 sequences represented by GB40_w2_1, two minor clusters, each with 4 sequences, represented by GB40_w2_13 and GB40_w2_17, respectively, and a single distal sequence (singlet) GB40_w2_15. Similarly, the 57 FF69_w2 sequences revealed five clusters and five singlets, with two major clusters of 27 and 16 sequences, represented by clones FF69_w2_27 and FF69_w2_52, and then FF69_w2_17 and FF69_w2_60, respectively. The three minor clusters each contained 3 sequences, of which FF69_w2_91 was cloned. Of the five FF69_w2 *env* singlets, FF69_w2_12 was cloned. These data indicated that multiple variants were transmitted and successfully established acute systemic infections in GB40 and FF69, with 1–2 dominant strains in each animal. Though GB40 and FF69 received the same single dose SHIV_SF162P3N_ intrarectal challenge, the dominant T/F strains in these animals were not the same. Of the 45 inoculum-derived V1–V5 sequences, which had a maximum distance of 3.98% and a mean distance of 2.42%, we did not identify any that was identical to the dominant GB40 or FF69 T/F strains, with only one sequence closely related to the second largest FF69_w2 cluster, represented by FF69_w2_17 and FF69_w2_60. Because the uncloned SHIV_SF162P3N_ was derived from an end stage infection (66 wpi), most variants might not be fit for transmission. Therefore, it was not surprising that minor variants in the inoculum were transmitted in GB40 and FF69. Additionally, in the phylogenetic tree, the vast majority of the FF69_w8 sequences were positioned in a separate branch between the two FF69_w2 dominant clusters, suggesting a likely viral recombination event in this animal.

### 3.3. Sequential Neutralizing Antibody Development and Neutralization Sensitivity of the T/F Env Strains

**Figure 3:**
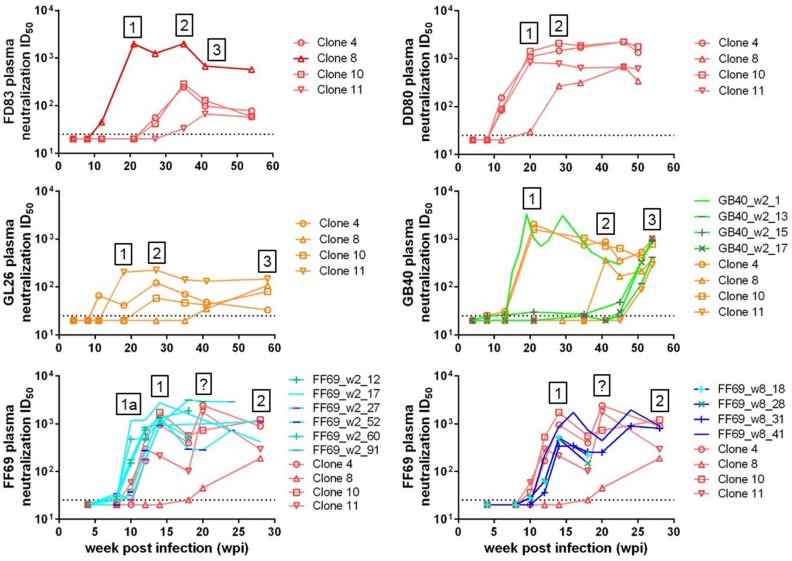
Longitudinal plasma neutralization ID_50_ titers against the SHIV_SF162P3N_ inoculum-derived clones 4, 8, 10 and 11, and against the autologous T/F or w8 viruses in macaques FD83, GB40 and FF69. Each line represents a tested Env-pseudovirus as indicated. Boxed numbers or question marks denote sequential waves or questionable waves of neutralizing antibody responses defined by the tested Env-pseudoviruses.

With known T/F viruses in animals FD83 (infected with clone 8), GB40 and FF69, we examined the initial autologous neutralizing antibody responses in these animals. Prior to that, from the longitudinal plasma neutralization titers against the inoculum-derived clones 4, 8, 10 and 11, we observed 2–3 sequential waves of neutralizing antibodies in all 5 macaques with breadth (Figure 3), with each wave defined as a new activity capable of neutralizing a prior resistant Env clone. Specifically, in FD83, the first wave (wave 1) of neutralizing antibodies appeared at w20 against the infecting molecular clone 8, followed by a second wave (wave 2) at w35 against clones 4 and 10, and then a third wave (wave 3) at w42 against clone 11. In DD80, wave 1 peaked at w20 against clones 4, 10 and 11, followed by wave 2 at w28 against clone 8. In the Chinese macaque GL26, wave 1 peaked at w18 against clone 11, followed by wave 2 at w27 against clones 4 and 10, and then wave 3 at w58 against clone 8. In another Chinese macaque GB40, wave 1 appeared around w21 against clones 4 and 10, followed by wave 2 at w42 against clone 8, and then wave 3 at w54 against clone 11. In FF69, wave 1 appeared early at w12–14 against clones 4, 10 and 11, followed by wave 2 at w28 against clone 8. Also in FF69, the neutralization ID_50_ titers for the wave 1 sensitive clones 4, 10 and 11 fluctuated—first dropping at w18 and then rising at w20—this pattern of change in titers might suggest a separate wave of neutralizing antibody development at w20, and thus we noted it with a question mark (Figure 3). Depending on the number and sequence of the Env clones tested, the number of neutralizing antibody waves might change. The 2–3 waves detected here might not represent all of the neutralizing antibodies in these animals; if more waves exist, those will need to be revealed by additional Env clones. The neutralization ID_50_ titers of the first wave were commonly over 1000, and the ID_50_ titers of waves 2–3 were more moderate in the range of hundreds. These data indicated that given time, the plasma neutralizing antibodies in these animals gradually broadened to cover more genetically diverse viral variants, though the titers may not reach as high as the initial wave 1 responses.

Unlike FD83 infected with a molecular clone (clone 8), GB40 and FF69 were infected with multiple T/F viruses, and thus we asked whether all of the T/F viruses were equal in sensitivity to autologous neutralization. To address this question, we cloned representative Env sequences from the T/F clusters or singlets and tested them for neutralization sensitivity to autologous longitudinal plasmas (Figure 3). A total of 4 GB40_w2 *env* sequences were cloned—namely, GB40_w2_1, 13, 15 and 17 (Table 1), each representing a T/F cluster or singlet (Figure 2). GB40_w2_1, representing the dominant cluster, was sensitive to the w19 plasma neutralization, with an ID_50_ > 1000, recapitulating the wave 1 neutralizing antibody activity in this animal (Figure 3). The other three GB40_w2 Env clones, however, were all resistant to the early plasmas and became sensitive to plasmas from w52 and later, recapitulating the wave 3 activity in this animal (Figure 3). This data suggested that although multiple variants were transmitted, it was the dominant GB40_w2_1 Env clone that initiated the wave 1 neutralizing antibody response in GB40. Similarly, we cloned 6 FF69_w2 *env* sequences—namely, FF69_w2_12, 17, 27, 52, 60 and 91 (Table 1), each representing a T/F cluster or singlet (Figure 2). All 6 FF69_w2 Env clones were sensitive to the w12–14 plasmas, with peak ID_50_ titers near or over 1000 (Figure 3). In terms of timing, the dominant cluster clones FF69_w2_27 and FF69_w2_52 recapitulated the wave 1 activity at w12–14; the second dominant cluster clones FF69_w2_17 and FF69_w2_60 defined an even earlier wave of activity at w10, of which we named wave 1a; the sensitivities of the minor clones FF69_w2_12 and FF69_w2_91 arose in between. Therefore, in contrast to the GB40 T/F variants, all 6 tested FF69 T/F variants appeared contribute to the initial wave 1a and wave 1 responses—in this case, it is our opinion that more weight should be given to the two dominant clusters based on their greater antigen loads than those of the minor variants. From the 54 FF69_w8 *env* sequences, we cloned and tested 4 Envs—namely, FF69_w8_18, 28, 31 and 41 (Table 1 and Figure 2). All 4 FF69_w8 Env clones were sensitive to the w12–14 plasmas, recapitulating the wave 1 activity in this animal, with FF69_w8_41 being the most sensitive (Figure 3). These data indicated that the FF69_w8 viral variants have not yet escaped wave 1 neutralizing antibodies, which did not arise until w12–14.

### 3.4. Targets of Wave 1 Neutralizing Antibodies in SHIV-Infected Macaques

**Figure 4:**
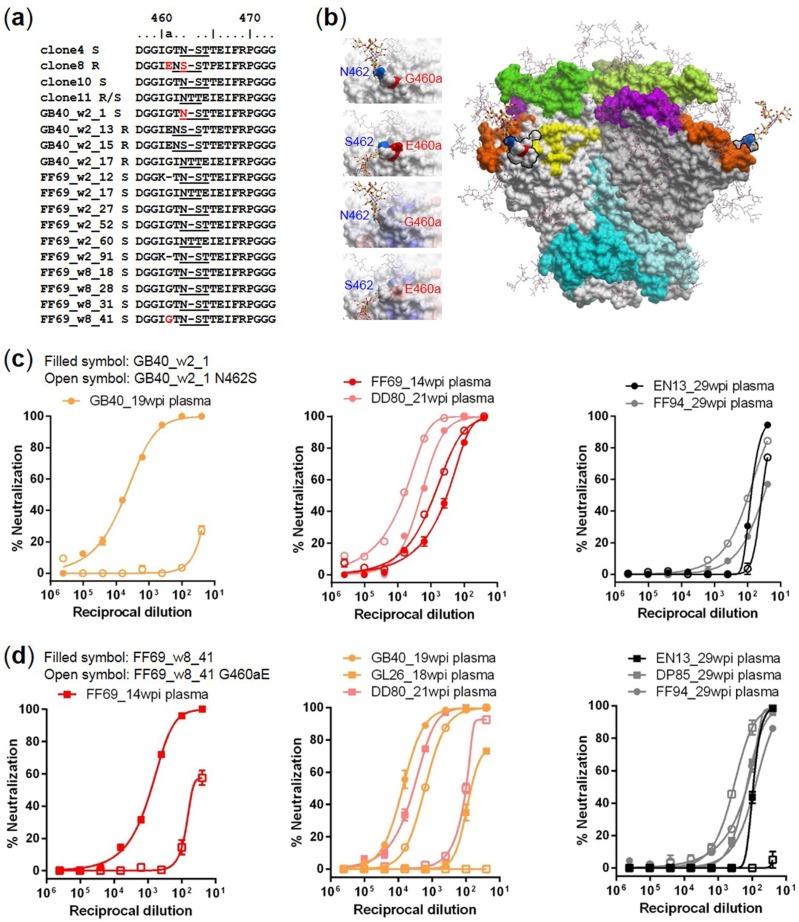
The initial autologous neutralizing antibody responses in SHIV_SF162P3N_ infected macaques GB40 and FF69 targeted the gp120 V5. (**a**) Alignment of the V5 amino acid sequences of the tested Env clones indicated with “S” for neutralization sensitivity, “R” for resistance and “R/S” for resistance to GB40 wave 1 but sensitivity to FF69 wave 1. Underlines indicate putative *N*-linked glycosylation sites; “-” indicate gaps. Mutations N462S and G460aE are highlighted in red, each in a sensitive and a resistant clone; (**b**) Modeling of N462S and G460aE mutations on the JR-FL SOSIP structure (PDB: 5FYK) with side chains optimized. In the trimer structure, gp120 (grey) and gp41 (cyan) are displaced as surfaces with glycans as sticks (two visible N462 glycans are thicker than other glycans), and regions of V1V2 (green), V3 (magenta), V4 (orange) and CD4-binding site (yellow) are highlighted, while V5 is outlined with black lines. Residues 460a and 462 are colored red and blue, respectively. Left small panels: detailed N462S and G460aE changes in glycan location and side chains (top two panels) and in electrostatic potential surfaces (bottom two panels); (**c**) Neutralization profiles of GB40_w2_1 wild-type (filled symbol) versus its N462S mutant (open symbol) and (**d**) FF69_w8_41 wild-type (filled symbol) versus its G460aE mutant (open symbol), using early plasmas from 14 to 29 wpi as indicated from autologous animals GB40 and FF69 (**left**), homologous animals with neutralization breadth (**middle**), and homologous animals without neutralization breadth (**right**).

To map the targets of wave 1 neutralizing antibodies, we aligned the amino acid sequences of the tested Env clones and identified two mutations in gp120 V5 that were associated with changes in neutralization sensitivity, N462S for GB40_w2_1 and G460aE for FF69_w2 and FF69_w8 clones (Figure 4a). Note that N462 is a putative *N*-linked glycosylation site, and the N462S mutation has been associated with the N462 glycan shifting to N461 in the resistant clones. Modeling of the N462 and G460a on a JR-FL SOSIP trimer structure (PDB: 5FYK) [39] indicated that both residues were exposed on the surface, and that the orientation of the N462 glycan could be very different when shifted to N461 (Figure 4b). Also, the G460aE mutation introduced a bulkier side chain and a negatively charged surface from a neutral surface in the center of the region (Figure 4b). Therefore, both mutations were likely to significantly alter the potential antibody interacting surface of the V5 region.

In FD83, because the infecting virus was clone 8, which has naturally S462 and E460a residues (Figure 4a) and is resistant to both GB40 and FF69 wave 1 neutralization (Figure 3), the N462 glycan and G460a cannot be the targets for FD83 wave 1 antibodies. We constructed the GB40_w2_1 N462S mutant and compared its neutralization sensitivity to wild-type (Figure 4c). The N462S mutation rendered the sensitive GB40_w2_1 clone resistant to GB40 wave 1 neutralization, as assessed by the GB40_19wpi plasma (Figure 4c, left), indicating that GB40 wave 1 targeted the N462 glycan. However, other macaques with breadth did not seem to share this target. Excluding FD83 and GL26, of which the early plasmas did not neutralize GB40_w2_1, both FF69 and DD80 wave 1 plasmas neutralized GB40_w2_1, but the neutralization sensitivities were not reduced upon the N462S mutation (Figure 4c, middle). Two macaques without breadth, EN13 and FF94, also neutralized GB40_w2_1 (Figure 4c, right); the EN13_29wpi plasma was moderately sensitive to N462S, and the FF94_29wpi plasma was not sensitive. Likewise, we constructed the FF69_w8_41 G460aE mutant and compared its neutralization sensitivity to wild-type (Figure 4d). The G460aE mutation largely reduced the neutralization sensitivity of FF69_w8_41 to FF69 wave 1, as assessed by the FF69_14wpi plasma (Figure 4d, left), indicating that FF69 wave 1 targeted G460a. In contrast to N462 in GB40, the G460a target was shared by other macaques with breadth, including GB40. Excluding FD83, the wave 1 plasmas of GB40, GL26 and DD80 all neutralized FF69_w8_41, and their neutralization sensitivities were clearly reduced upon the G460aE mutation, with the reduction of GB40_19wpi plasma neutralization being the modest (Figure 4d, middle). Three macaques without breadth, EN13, DP85 and FF94, also neutralized FF69_w8_41 (Figure 4d, right); the EN13_29wpi plasma was sensitive to the G460aE mutation, and the DP85_29wpi and FF94_29wpi plasmas were not.

## 4. Discussion

In the context of SHIV_SF162P3N_ infection, with 5 macaques that developed neutralization breadth and 6 macaques that did not, we were first interested in potential virological and immunological factors that distinguish them. We compared their plasma viral load and blood CD4 count and found that these factors did not distinguish between the two groups. This result was unable to confirm the finding of an association between higher plasma viral load and lower blood CD4 count with bnAb development from a previous study of 40 HIV-1 clade C infected women [40]. However, the association may be too marginal to be detected by the small number of animals analyzed here. We next assessed 4 homologous Env clones for any association between early (typically also potent) homologous neutralization with later breadth development and found only with clone 10 a trend for macaques with breadth favoring an early homologous response. Our interpretation of this result was that an early and potent homologous neutralization response was probably essential but not sufficient for later breadth development.

We were next interested in characterizing the initial autologous neutralizing antibody responses in three macaques that developed neutralization breadth, FD83, GB40 and FF69. As FD83 was infected with a molecular clone (clone 8), we examined the T/F viruses in GB40 and FF69 infected with uncloned SHIV_SF162P3N_. In contrast to HIV-1 transmission typically with only a single T/F virus establishing infection in the human host [41,42,43], the single dose intrarectal SHIV_SF162P3N_ infection model, as demonstrated in GB40 and FF69, resulted in multiple viral transmissions with 1–2 dominant T/F variants. We then asked whether the T/F viruses were equal in sensitivity to autologous plasma neutralization. In GB40, only the dominant T/F strain, represented by GB40_w2_1, was sensitive; however, in FF69, all tested T/F variants were sensitive, differing slightly in timing to reach peak titers. Therefore, when transmission occurred with only a single variant, the T/F variant was likely to stimulate the initial autologous neutralizing antibody response; when transmission occurred with multiple variants, as demonstrated in GB40 and FF69, both single and multiple variants could contribute to the elicitation of neutralizing antibodies. In terms of timing, the initial autologous neutralization reached peak titers between 10 and 20 wpi in these animals, which was in general consistent to the timing of 2–6 mpi for autologous neutralizing antibody development reported in HIV-1 infected humans. Because FF69 developed the earliest autologous neutralizing antibodies at 10 wpi among the studied animals, we speculate that this early response may have resulted from the multiple T/F variants rather than a single T/F variant stimulating the response.

We further mapped in gp120 V5 two mutations, N462S and G460aE, largely responsible for wave 1 neutralization escape in GB40 and FF69, respectively. Because the T/F virus in FD83 (with breadth) and FF94 (without breadth) was clone 8, which has naturally S462 and E460a residues and was resistant to GB40 and FF69 wave 1 neutralization, the N462 glycan and G460a could not be the target for FD83 and FF94 and thus was irrelevant to these animals. The N462 glycan target identified in GB40 was not shared in two other macaques with breadth (FF69 and DD80) but was partially shared in macaque EN13 that did not develop breadth. In contrast, the G460a target identified in FF69 was shared in three other macaques with breadth (GB40, GL26 and DD80) and also shared in EN13 that did not develop breadth but not shared in another macaque DP85 without breadth. Taken together, although it is unclear whether N462 or G460a is related to later neutralization breadth development, G460a but not N462 appeared more common in macaques with breadth than those without. Similarly to the targets reported in HIV-1 infected humans [16,17], these sites were located in a variable loop of gp120, potentially explaining the antibody’s narrow specificity. Nevertheless, these sites have not been reported previously and were different from the targets reported to date in HIV-1 infected humans.

Finally, longitudinal assessment of plasma neutralization against 4 inoculum-derived Env clones revealed 2–3 sequential waves of neutralizing antibodies in macaques with breadth, exhibiting higher titers for wave 1 and lower titers for waves 2–3, possibly due to many more antibody lineages neutralizing the initial Env strains for wave 1 and very few antibody lineages in waves 2–3 neutralizing a range of genetically diverse Env strains, indicative of breadth. This observation supported the current model that viral escape and gradually increased Env diversity drives the maturation and development of bnAb lineages. Our study has only identified the autologous Env clones as putative antigens for the first wave; to capture the full course of bnAb development in these animals, future studies will need to identify sequential autologous Env clones that are responsible for wave 2–3 antibodies.

Our results have implications for HIV-1 vaccine development. Though it is recognized that a single Env immunogen is unlikely to induce bnAbs and that sequential Env immunization is likely required [44,45,46,47], it is unclear how many Envs are needed and what Env sequences should be used. Using the SHIV_SF162P3N_ infected macaques studied here as templates, one could first attempt to reproduce their antibody responses, either with SHIV infection or Env immunization. Based on the observation that the studied animals each developed 2–3 sequential waves of neutralizing antibodies, we estimate that 3 sequential Envs, if not more, would be needed to drive bnAb maturation. In terms of selecting Env sequences for immunogens, it is our view that the autologous Env clones responsible for each wave of neutralizing antibody responses should be considered.

## Figures and Tables

**Table 1:** Summary of SHIV_SF162P3N_ *env* sequences isolated and tested in this study.

Macaque ID	Time Point	Sample Type	CD4 Count Cells/μL	Plasma vRNA Copies/mL	No. of Env SGA	Mean ± SD Distance (%)	Maximum Distance (%)	No. of Env Clones	Env Clones	Wave 1 Neutralization
GB40	2 wpi	Plasma	1904	4,276,200	22	0.74 ± 0.63	2.12	4	w2_1	Sensitive
									w2_13,15,17	Resistant
FF69	2 wpi	Plasma	269	37,524,600	57	1.01 ± 0.62	2.17	6	w2_12,17,27,52,60,91	Sensitive
FF69	8 wpi	Plasma	348	775,130	54	1.15 ± 0.36	2.33	4	w8_18,28,31,41	Sensitive
